# Emerging immunotherapies for metastasis

**DOI:** 10.1038/s41416-020-01160-5

**Published:** 2020-12-02

**Authors:** Sarah C. Edwards, Wilma H. M. Hoevenaar, Seth B. Coffelt

**Affiliations:** 1 Cancer Research UK Beatson Institute, Glasgow, UK; 2 Institute of Cancer Sciences, University of Glasgow, Glasgow, UK

**Keywords:** Tumour immunology, Immunotherapy, Tumour immunology

## Abstract

Major advances in cancer immunotherapy have dramatically expanded the potential to manipulate immune cells in cancer patients with metastatic disease to counteract cancer spread and extend patient lifespan. One of the most successful types of immunotherapy is the immune checkpoint inhibitors, such as anti-CTLA-4 and anti-PD-1, that keep anti-tumour T cells active. However, not every patient with metastatic disease benefits from this class of drugs and patients often develop resistance to these therapies over time. Tremendous research effort is now underway to uncover new immunotherapeutic targets that can be used in patients who are refractory to anti-CTLA-4 or anti-PD-1 treatment. Here, we discuss results from experimental model systems demonstrating that modulating the immune response can negatively affect metastasis formation. We focus on molecules that boost anti-tumour immune cells and opportunities to block immunosuppression, as well as cell-based therapies with enhanced tumour recognition properties for solid tumours. We also present a list of challenges in treating metastatic disease with immunotherapy that must be considered in order to move laboratory observations into clinical practice and maximise patient benefit.

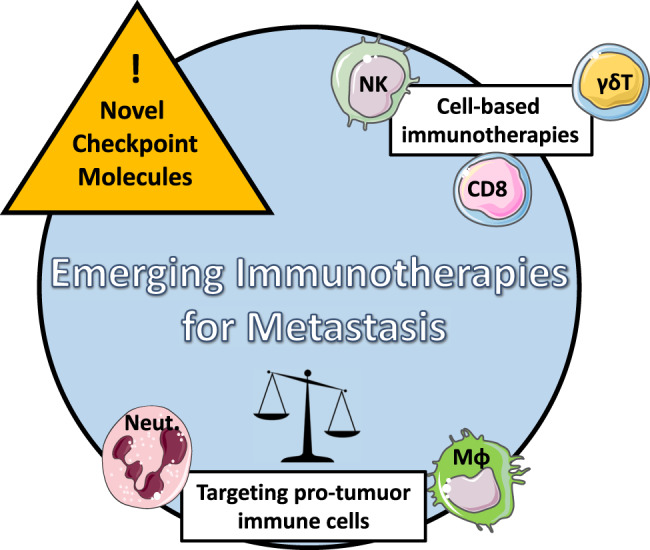

## Background

Cancer cells can detach from the primary tumour, intravasate into the blood or lymphatic system and migrate to distant sites where they extravasate from the blood or lymph vessels to seed secondary sites. Controlling the spread of cancer and outgrowth at these secondary sites remains the most challenging aspect of oncology. Across all cancer types, only around 20% of patients with stage IV metastatic disease survive beyond 5 years of diagnosis.^1^ Advances in immunotherapy have started to reverse these dire statistics and, because of their success in some types of metastatic cancer, immunotherapies have been heralded as revolutionary drugs in the treatment of metastatic disease. T-cell checkpoint inhibitors, in particular, such as those that target programmed cell death protein 1 (PD-1), its ligand PD-L1, or cytotoxic T-lymphocyte-associated protein 4 (CTLA-4), are responsible for the majority of immunotherapy successes in cancers such as melanoma and lung cancer; other cancer types exhibit lower numbers of responders with these drugs, which could be due to the lack of T-cell infiltration or (neo)antigen expression.^2^ Manipulating a cancer patient’s own T cells has solidified the concept that immune cells can be targeted effectively for patient benefit. However, cancer immunotherapy has a limited range. We have already learnt from clinical trials testing anti-CTLA-4 and anti-PD1 that these drugs are largely futile in eradicating secondary tumours and extending the lifespan of most cancer patients with metastasis, due to inherent or acquired drug resistance.^3^ Consequently, alternative immunotherapeutic approaches are required to counteract metastasis in patients that are unresponsive to CTLA-4 and/or PD-1/PD-L1 inhibitors. As invasion and metastasis rely heavily on the pro-tumour and anti-tumour functions of immune cells, understanding the cellular and molecular processes that underpin the progression of metastatic disease is likely to offer novel immunotherapy options. In this review, we discuss the advances that have been made in generating immunotherapeutic alternatives to CTLA-4 and PD-1/PD-L1 in experimental model systems of solid tumours, as well as highlighting the challenges in treating secondary tumours with immunotherapy.

## Enhancing endogenous anti-tumour immunity

**Fig. 1:**
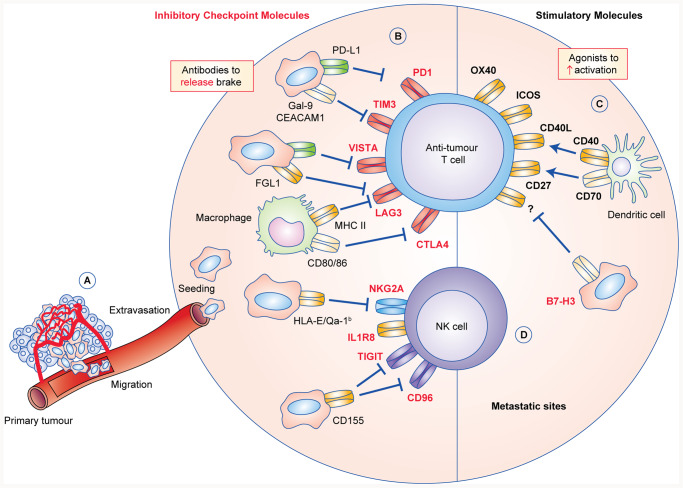
Inhibitory and stimulatory immune checkpoint molecules regulate the anti-tumour response at metastatic sites. **a** In metastatic progression, cancer cells detach from the primary tumour, intravasate into the blood or lymphatic system and migrate to distant sites where they extravasate from the blood or lymph vessels to seed secondary tumour sites. **b** Cancer cells, metastasis-associated macrophages and other cells at metastatic sites can express a plethora of immunomodulatory proteins to inhibit and activate anti-tumour T cells. The binding of these ligands to their cognate checkpoint receptors, such as programmed death-ligand 1 (PD-L1) with programmed cell death protein 1 (PD-1) and galectin-9 or carcinoembryonic antigen-related cell adhesion molecule 1 (CEACAM1) with TIM-3, dampens T-cell activation and effector anti-tumour T-cell responses. Checkpoint molecules, such as VISTA, LAG-3 and CTLA-4 are inhibitory receptors that deliver negative stimulation signals upon binding to MHC-II, FGL1 and the co-stimulation molecules CD80 and CD86. **c** Engagement of stimulatory receptors such as OX40, ICOS, CD40, B7-H3 and CD27 with their cognate ligand, or agonists that artificially provide these signals, drives T-cell activation, differentiation and effector responses (R? = unknown receptor). Dendritic cells can be activated through CD40 and CD70 to induce their maturation and antigen-presenting properties. **d** Natural killer (NK) cells can be manipulated by cancer cells and myeloid cells that express inhibitory ligands to dampen their cytotoxic effector responses. The inhibitory receptors T-cell immunoreceptor with Ig and ITIM domains (TIGIT) and CD96 both have affinity for CD155, which is expressed on many types of cancer cell. NKG2A binds HLA-E on human cells or Qa-1^b^ on mouse cells to block NK-cell-mediated killing. These inhibitory and activatory checkpoint pathways can be selectively modulated by blocking or agonist antibodies to release the brake on anti-tumour immunity in order to treat or prevent metastatic disease.

In addition to CTLA-4 and PD-1, a number of other T-cell checkpoint inhibitors that might prove extremely valuable in treating metastatic tumours have emerged over the past years, such as TIM-3 and LAG-3 (Fig. 1).^4^ Furthermore, the catalogue of inhibitory molecules has expanded to include receptors expressed on natural killer (NK) cells,^5^ several of which are shared by γδ T cells.^6^ Given that NK cells are critical in combating metastasis,^7^ blocking inhibitory molecules on NK cells, which limit their killing and cytotoxic molecule production, such as TIGIT and NKG2A, could prove very useful in treating patients with metastatic disease. Below we outline such T-cell-associated immune checkpoint inhibitors and NK cell-associated immune checkpoint inhibitors. Employing alternative immune checkpoint inhibitors to anti-CTLA-4 and anti-PD-1 that activate innate-like and/or adaptive lymphocytes could provide additional benefit or perhaps even function more effectively than the standard anti-CTLA-4/PD-1 therapies (Fig. 1).

### TIM-3

One of the most promising immune-modulating checkpoints currently under investigation is T-cell immunoglobulin and mucin domain 3 (TIM-3), encoded by the gene *HAVCR2.*^8^ TIM-3 can be expressed by multiple subsets of T cells including CD8^+^ and CD4^+^ T cells, regulatory T cells and NK cells,^9–11^ as well as myeloid cells, such as dendritic cells and macrophages.^12–15^ In the absence of ligand, TIM-3 recruits the tyrosine kinase LCK to mediate T-cell activation. However, ligand engagement of TIM-3 is known to disrupt the immunological synapse, interfere with LCK signalling and induce T-cell apoptosis (reviewed in ref. ^8^). The two most well-studied ligands of TIM-3 include galectin-9 and carcinoembryonic antigen-related cell adhesion molecule 1 (CEACAM1), both of which can be produced by cancer cells and myeloid cells (reviewed in ref. ^8^).

The expression of TIM-3 is associated with advanced tumour stage and lymph node metastasis in lung cancer patients.^10^ In patients with metastatic melanoma, TIM-3 expression is also associated with dysfunctional/exhausted CD8^+^ T cells and NK cells, and the inhibition of TIM-3 signalling ex vivo increases the functional capacity of these cells.^16,17^ Interestingly, the reversion of patient-derived CD8^+^ T-cell dysfunction requires dual blockade of both TIM-3 and PD-1.^16^ After anti-PD-1 therapy, the expression of TIM-3 increases on CD4^+^ and CD8^+^ T cells in metastatic lung cancer patients and lung cancer mouse models driven by mutant epidermal growth factor receptor (EGFR) or mutant KRAS,^18^ suggesting that this molecule is involved in adaptive resistance to PD-1 inhibition. Indeed, the combinatorial treatment of lung-tumour-bearing *CC10-rtTA;Tre-Egfr*^T790M/L858R^ mice with anti-PD-1 and anti-TIM-3 extends survival compared with anti-PD-1 blockade alone.^18^ Similarly, targeting both PD-1 and TIM-3 in mice bearing transplantable CT26 or MC38 colorectal cancer cells or B16 melanoma cells slows primary tumour growth.^19^ In the *MMTV-PyMT* mammary tumour model, paclitaxel and anti-TIM-3 blocking antibodies control primary tumour growth more efficiently than chemotherapy alone, while anti-TIM-3 monotherapy is ineffective.^15^ Mammary tumour-infiltrating CD103^+^ dendritic cells express high levels of TIM-3, whereas the expression of TIM-3 in CD8^+^ T cells is negligible in this model. These TIM-3-expressing CD103^+^ dendritic cells play a crucial role in the chemotherapy response, as their depletion abrogates the response to paclitaxel/anti-TIM-3 treatment. The blockade of TIM-3 or galectin-9 in *MMTV-PyMT* tumour-bearing mice increases the expression of CXC chemokine ligand 9 (CXCL9) in CD103^+^ dendritic cells, which recruits cytotoxic CXC chemokine receptor 3 (CXCR3)^+^ CD8^+^ T cells to tumours.^15^ Given the importance of TIM-3-expressing T cells and dendritic cells in cancer progression and chemotherapy response, TIM-3 inhibition might be useful in the metastatic setting to recruit cytotoxic T cells or to reinvigorate exhausted T cells in immunotherapy-naïve and/or anti-PD-1 refractory patients.

### LAG-3

Lymphocyte activation gene 3 (LAG-3) is another checkpoint molecule expressed on T cells and NK cells; it exerts its inhibitory function by binding to MHC class II and other ligands, such as LSECtin (reviewed in ref. ^20^). A newly discovered LAG-3 ligand produced by liver and cancer cells—fibrinogen-like protein 1 (FGL1)—has been identified to mediate antigen-specific T-cell suppression.^21^ Increased LAG-3 expression on CD4^+^ and CD8^+^ T cells is associated with liver metastasis in mismatch-repair-proficient colorectal cancer. Like PD-1 and TIM-3, LAG-3 expression is associated with dysfunctional tumour-infiltrating T cells in mouse models and human metastatic tumours,^22–24^ and the combination of anti-PD-1 and anti-LAG-3 treatment delays tumour growth in mice bearing subcutaneous Sa1N fibrosarcoma cells or MC38 colorectal cancer cells.^25^ The anti-PD-1/anti-LAG-3 combination is also highly efficacious in the metastatic IE9mp1 transplantable ovarian cancer model.^26,27^ In lung colonisation models of experimental metastasis using 4T1 mammary cells, LAG-3 blockade accompanied by treatment with immunostimulatory interleukin (IL)-12 reduces tumour growth more effectively than anti-LAG-3 or IL-12 treatment alone. In this model, tumour control is mediated by targeting LAG-3-expressing NK cells with anti-LAG-3 antibodies.^28^

The expression of LAG-3 is regulated by glycogen synthase kinase-3 (GSK-3), and attempts at decreasing LAG-3 transcription in T cells and NK cells using a GSK-3β inhibitor have shown that this is a viable strategy to counteract B16 melanoma growth in the lung.^29^ In another approach, instead of blocking LAG-3 signalling, LAG-3 fused to the Fc region of IgG1 (LAG-3Ig or IMP321) can be used as an MHC-II agonist to activate dendritic cells and anti-tumour T-cell responses through so-called immunopotentiation. An early phase trial with metastatic breast cancer patients demonstrated the potency of this molecule in combination with paclitaxel, where 15 out of 30 women exhibited an objective tumour response.^30^ Thus, manipulating LAG-3 in patients with metastasis shows promise.

### B7-CD28 superfamily members

CTLA-4 and PD-1 belong to the B7-CD28 superfamily. Some of the lesser-known members of this family play a role in primary tumour progression, suggesting that there could be benefit in targeting these molecules in the metastatic setting. For example, stimulation of inducible T-cell co-stimulator (ICOS) is required for the anti-tumour efficacy of anti-CTLA-4 in B16 and MC38 primary tumours, by generating an effector T helper 1 (T_H_1)-like population that plays a role in limiting tumour growth.^31–33^ Targeting V-domain Ig suppressor of T-cell activation (VISTA), which can be expressed by cancer cells or antigen-presenting cells, delays tumour growth in transplantable models and the transgenic *Tyr::Cre*^ERT2^*;Braf*^V600E^*;Pten*^F/F^ melanoma model, and shifts the tumour microenvironment towards anti-tumour immunity.^34,35^ B7-H3 (also known as CD276) is expressed on cancer cells and tumour-associated endothelial cells. Targeting B7-H3 with antibody–drug conjugates reduces metastatic progression, and this effect is independent of adaptive immune cells, as nude mice were used in these experiments.^36^ Further experimentation of inhibitors to these molecules in metastasis models is required to understand whether their function is similar between primary and secondary tumours.

### TNFR superfamily members

Some data on the importance of the tumour necrosis factor receptor (TNFR) superfamily in promoting or restricting immunity to tumours exist.^37^ This group of molecules comprises largely co-stimulatory molecules that synergise with T-cell receptor signalling to promote T-cell division. For example, OX40 is upregulated on activated CD4^+^ and CD8^+^ T cells, and an agonistic antibody of OX40 synergises with an inhibitor of transforming growth factor (TGF)-β to reduce the primary tumour growth of 4T1 mammary cells as well as spontaneous lung metastasis.^38^

CD4^+^ T cells activate CD40 on antigen-presenting cells, to facilitate the maturation of these cells, and agonists to CD40 have been used to stimulate CD103^+^ dendritic cells and prime tumour-specific T cells in genetically engineered mouse models of pancreatic ductal adenocarcinoma.^39,40^ CD40 agonist therapy—either as monotherapy or in combination with cytokines or anti-PD-1 or agonists of the TNFR member CD137—counteracts metastasis in transplantable melanoma, pancreatic, colon and kidney cancer models,^41–44^ and this immunotherapy can re-polarise myeloid cells towards an anti-tumour phenotype.^41,44^ Similarly, agonists of another TNFR member, CD27, reduce lung tumour burden of intravenously injected B16 melanoma cells.^45^ Naïve CD4^+^ and CD8^+^ T cells constitutively express CD27, and its activation by the ligand CD70 on dendritic cells supports T-cell priming. CD27 signalling has been found to be necessary to generate robust cytotoxic T cells,^46,47^ so CD27 agonists might further improve anti-PD-1 or CTLA-4 immunotherapy in cases where tumour-specific T cells are suboptimal. Other TNFR superfamily members, such as CD30, GITR, BTLA, are currently not well studied in the metastatic setting.

### TIGIT and CD96

T-cell immunoreceptor with Ig and ITIM domains (TIGIT; a co-inhibitory receptor expressed by T cells and NK cells) and CD96 comprise a pathway analogous to CTLA-4 with CD28, where they bind the same interacting partner—CD155—to negatively regulate NK cell function [reviewed in^48^]. Inhibition of TIGIT with neutralising antibodies in lung colonisation experiments of 4T1 mammary cell lines or B16 melanoma cells or carrying out the experiments in TIGIT knockout mice reduces lung tumours and extends survival.^49^ One study found that anti-TIM-3 was required in *Tigit*^–/–^ mice to reduce experimental lung metastasis of B16 cells.^50^ CD8^+^ T cells also express TIGIT,^51^ making it an ideal immunotherapy target to boost the anti-tumour functions of two cytotoxic cell types. Similarly, anti-CD96 therapy reduces 4T1 or B16 tumours in the lung and this effect is enhanced by the addition of anti-CTLA-4, anti-PD-1 or doxorubicin chemotherapy.^52^ Likewise, *Cd96*^–/–^ mice develop fewer experimental lung metastases than wild-type mice after tail vein injection of B16 cells, and this result is dependent on NK cells and interferon (IFN)γ.^53^ As both TIGIT and CD96 bind CD155, targeting both TIGIT and CD96 might be essential to achieve maximum anti-metastatic benefit.^52^

### NKG2A

NKG2A is another putative checkpoint inhibitor for NK cells and CD8^+^ T cells; its activation occurs through binding of HLA-E in humans or Qa-1^b^ in mice.^54^ Blocking NKG2A using the humanised anti-NKG2A antibody monalizumab increases NK-mediated killing through antibody-dependent cell-mediated cytotoxicity (ADCC) of mouse lymphoma cells in vivo; however, the potency of monalizumab in counteracting metastases of solid tumours remains to be seen.^55^ Clinical trials are underway examining monalizumab with cetuximab (anti-EGFR) in patients with metastatic colorectal or head and neck cancers (NCT026435509).

### IL-1R8

Interleukin 1 receptor 8 (IL-1R8, also called SIGIRR or TIR8) has been identified as a checkpoint inhibitor on NK cells. Spontaneous lung metastasis from MN/MCA1 sarcoma cells is reduced in *Il1r8*^−/−^ mice when compared with wild-type mice but primary tumour growth remains unaffected. Similarly, *Il1r8*^−/−^ mice are protected from liver metastasis of MC38 cells, and these events are reversed by NK cell depletion.^56^

## Cell-based immunotherapies for metastatic disease

T cells, NK cells and dendritic cells can be harvested from either cancer patients or healthy donors, expanded ex vivo, and then transfused back into cancer patients in the process of adoptive cell therapy (ACT). Whereas ACT using αβ T cells, γδ T cells or NK cells for the treatment of haematological malignancies is well documented,^5,57,58^ evidence for the use of such approaches to treat the metastatic disease of solid tumours is scant. This might be because of the time it takes to generate these cells, the lack of expression of specific tumour antigens or a lack of efficacy of the transplanted cells. However, a few examples of the potential of these strategies to treat metastatic disease do exist. Thus far, ACT of CD8^+^ T cells into metastatic melanoma patients has been demonstrated to be the most successful regimen among these types of immunotherapy (reviewed in ref. ^59^). Like checkpoint inhibitors, the efficacy of ACT might be dependent on high expression of (neo)antigens; however, effective ACT in breast cancer patients, whose tumours exhibit a much lower mutational burden, has been documented.^60^ One advantage of the innate-like lymphocytes, γδ T cells and NK cells is that they are not restricted by MHC molecules, which bypasses the importance of (neo)antigen expression. In fact, ACT of γδ T cells has induced complete remission of lung metastasis in a patient with renal cell carcinoma.^61^

Moreover, αβ T cells, γδ T cells and NK cells can also be genetically modified to enhance their anti-tumour properties, and several attempts have been made at harnessing the potent killing abilities of these cells by introducing transgenic T-cell receptors or chimeric antigen receptors (CARs) (reviewed in refs. ^57,62,63^). CAR-T cells are gaining traction in solid tumours and these cells might be useful for the metastatic disease if the correct antigen can be identified. For example, guanylyl cyclase C (GUCY2C)-targeted CAR-T cells can reduce CT26 colorectal cancer burden in the lungs of mice and extend survival when compared with CAR-T-cell control-treated mice.^64,65^ Human macrophages have also been engineered with CD3ζ-based CARs similar to T-cell CARs in order to direct the phagocytic activity of these cells against tumours, and these CAR-Ms reduce the burden of lung tumours by ovarian SKOV3 cancer cells.^66^ These HER2-directed CAR-Ms effectively reduced tumour burden and recruited T-cells and presented antigens to them. The field of cell-based immunotherapies is rapidly evolving, with various endeavours to make these products more specific, durable and safe, so that future versions are likely to improve their benefits in the metastatic setting.

## Inhibiting pro-tumour immune cells and immunosuppression at metastatic sites

Data from mouse metastasis models have highlighted a critical role for myeloid cells and some innate lymphocyte populations in metastatic progression. Here, we focus on monocytes/macrophages, neutrophils, regulatory T (T_REG_) cells and IL-17-producing γδ T cells. Since myeloid-derived suppressor cells (MDSCs) encompass both monocytes and neutrophils that are pathologically activated by tumour-derived factors to suppress anti-tumour immune cells,^67^ we refrain from using the MDSC nomenclature. Instead, we refer specifically to monocytes or neutrophils to be more precise about their individual role in metastasis formation. Information on other cell populations, such as eosinophils, basophils, mast cells and innate lymphoid cells (ILCs), is scarce in the metastatic setting, prohibiting a lengthy discussion on opportunities to target these cells. Further knowledge on these lesser-studied immune cells will expand the potential of targetable pathways. For example, a recent study found that group 2 ILCs and eosinophils support lung metastasis through suppression of NK cells, highlighting the cytokines, IL-33 and IL-5 in this process.^68^ While this section focuses on the pro-metastatic role of immune cells, it should be noted that the role of these cells in cancer progression is dynamic and subject to their local microenvironment. Immune cells, in particular macrophages and neutrophils, are not always pro-tumour – their function depends on their location, polarisation, maturation status and stage of the disease.

**Fig. 2:**
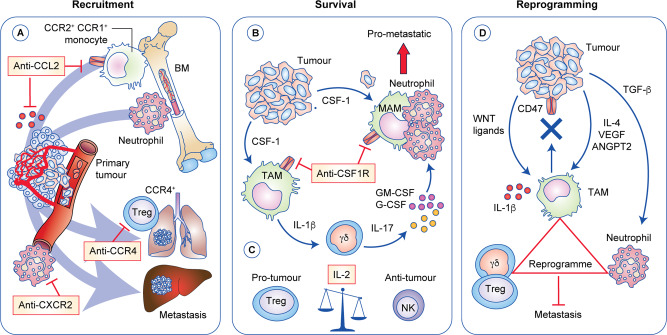
Exploiting pro-metastatic immune cell recruitment, survival and re-programming to counteract metastasis. Recruitment, survival and re-programming of immune cells to a pro-tumorigenic phenotype at distant sites are key processes in the metastatic cascade. **a** Primary and secondary tumours release chemokines that attract aiding and abetting immune cells to encourage metastasis. In many cancers, CCR2^+^ bone-marrow-derived monocytes are recruited to primary and secondary tumours by the chemokine ligand CCL2, where these monocytes differentiate into tumour-associated macrophages (TAM). Pro-metastatic CXCR2^+^ neutrophils are recruited by CXCL1, CXCL2 or CXCL5, while pro-tumour CCR4^+^ regulatory T (T_REG_) cells require CCL17 or CCL22. Targeting these chemokine pathways can prevent the accumulation of these cell types and reduce metastasis in the liver or lung of colorectal, pancreatic and breast cancer mouse models. **b** Targeting colony stimulating factor (CSF)-1, granulocyte-macrophage (GM)-CSF and granulocyte (G)-CSF affects the pro-metastatic cascade. TAMs secrete interleukin (IL)-1β to activate IL-17-producing γδ T cells, which induce immunosuppressive neutrophils through G-CSF. Metastasis-associated macrophages (MAM) provide growth factors, survival signals and angiogenic factors at secondary sites to support outgrowth of cancer cells. **c** The cytokine IL-2 is essential for the survival of pro-tumour T_REG_ cells as well as the activation of anti-tumour natural killer (NK) cells. Targeting selective IL-2 receptors on T_REG_ cells might prevent their accumulation while enabling anti-tumour NK cells to remain active. **d** Tumour-derived factors such IL-4, vascular endothelial growth factor (VEGF) and angiopoietin 2 (ANGPT2) can induce pro-tumorigenic macrophages, while transforming growth factor (TGF)-β can enhance pro-metastatic neutrophils. CD47 functions as a ‘don’t eat me’ signal and can be upregulated on metastatic cancer cells to evade immune surveillance and phagocytosis by macrophages. Tumour-derived WNT ligands induce macrophages to secrete IL-1β, which activates IL-17-producing γδ T cells to drive pro-metastatic neutrophils. Blocking or interfering with the cytokine cascade or receptors on these pro-metastatic immune cells could reprogramme them away from a pro-tumorigenic phenotype in order to prevent metastatic disease.

Macrophages are the best-studied group of immune cells in metastasis. We have known about their potent ability to support metastasis^69^ through angiogenesis^70,71^ for nearly 20 years, but these cells drive metastasis via multiple other mechanisms, such as providing growth factors for disseminated cancer cells as well as immune suppression of anti-tumour T cells and NK cells (reviewed in ref. ^72^). Neutrophils are increasingly being recognised for their pro-metastatic functions. These cells have been somewhat overlooked or avoided as they are difficult to manipulate, despite evidence of their involvement in metastasis existing more than 10 years before the data on pro-metastatic macrophages.^73^ Like macrophages, neutrophils can drive metastasis both from the primary tumour site or secondary locations before and after the arrival of disseminated cancer cells in distant organs (reviewed in ref. ^74^). Although low in number, γδ T cells can be very influential in cancer progression, where they orchestrate immune responses and modulate endothelial cells at metastatic sites primarily through the production of the pro-inflammatory cytokine IL-17.^75,76^ Macrophages, IL-17-producing γδ T cells and neutrophils can even work together to establish a systemic inflammatory pathway that suppresses CD8^+^ T cells at the pre-metastatic site and supports metastasis in p53-deficient mammary tumour models.^75,77^ Finally, T_REG_ cells are known to facilitate metastatic progression by immunosuppression, and to shield cancer cells from immune detection (reviewed in ref. ^78^). To interfere with the activity of these pro-metastatic immune cells, three key processes that can be targeted have been identified from preclinical studies: recruitment, survival and reprogramming (Fig. 2).^72^

### Blocking recruitment of pro-metastatic immune cells

CCR2^+^ bone marrow-derived monocytes are readily recruited to primary and secondary tumours in multiple tumour types by the chemokine CCL2.^79–81^ Consequently, the CCL2–CCR2 axis represents one point at which the accumulation of metastasis-associated macrophages (MAM) could be prevented.^82,83^ CCR2 small molecule antagonists are effective in transplantable models of pancreatic cancer and hepatocellular carcinoma,^80,84^ suggesting that secondary tumours may also be susceptible to these drugs. As pro-metastatic γδ T cells also express CCR2,^83,85,86^ inhibitors of CCL2 or CCR2 could be beneficial for targeting cancer types that rely on monocytes or γδ T cells or both. In lung metastasis of *MMTV-PyMT* tumours, CCR2 signalling regulates monocyte retention by CCL3 activation of CCR1,^87^ and CCR1^+^ cells are also important in driving colorectal cancer liver metastasis,^88^ highlighting another point of intervention to thwart metastasis. CCR5 is a another promising target for macrophages (and cancer cells) in colorectal cancer liver metastasis, as its inhibition repolarises macrophages towards an anti-tumoural phenotype.^89^ In a Phase 1 trial called MARACON, CCR5 inhibition with the HIV drug, maraviroc, in patients with metastatic colorectal cancer was well tolerated, and tumours exhibited reduced proliferation.^89^ However, there are risks to targeting chemokines, and the duration of treatment is critical to avoid a rebound effect that leads to increased metastasis, as is seen in models of breast cancer lung metastasis after anti-CCL2 therapy, where interruption of treatment releases monocytes from the bone marrow and accelerates metastasis formation.^90^ A new mouse model in which the chemokine receptors CCR1, CCR2, CCR3 and CCR5 are deleted together has been generated.^91^ The use of such models in combination with metastasis models will hopefully shed light on combinatorial chemokine receptor function for pro-metastatic immune cells and will help to determine the context in which to target these receptors.

Neutrophils use a different set of chemokine receptors to monocytes/macrophages, such as CXCR1 and CXCR2. Inhibition of CXCR2 in models of spontaneous metastasis—such as the *Kras*^G12D^*;Trp53*^R172H^*;Pdx1-Cre* (KPC) pancreatic cancer model or the *Villin-Cre*^*ER*^;*Kras*^G12D^*;Trp53*^F/F^*;Rosa26*^N1cd/+^ (KPN) colon cancer model—reduces the occurrence of secondary tumours in the liver without affecting survival.^92,93^ In the context of liver metastasis as well as lung metastasis,^75^ neutrophils suppress CD8^+^ T-cell responses to help disseminated cancer cells evade anti-tumour immunity, suggesting that combining neutrophil targeting with T-cell-based immunotherapy might be better than either approach alone. Indeed, treatment of pancreatic-tumour-bearing KPC mice with CXCR2 inhibitors and anti-PD-1 antibodies extends survival beyond monotherapy controls.^92^

Primary 4T1 mammary tumours can induce the production of CCL17/TARC (thymus- and activation-regulated chemokine) in the pre-metastatic lung, which guides the recruitment of CCR4^+^ T_REG_ cells and cancer cells to this site.^94^ The T_REG_ cells then protect cancer cells by inhibiting NK cells, thereby facilitating metastasis formation. Depleting T_REG_ cells, inhibiting CCR4 and the combined silencing of CCL17 and the T_REG_ master transcription factor FOXP3 in CCR4^+^ cells reduces the number of metastatic foci in the lung.^94^ Another way to reduce the recruitment of T_REG_ cells to pre-metastatic sites in liver and mammary tumour models is accomplished by reducing CCL22 secretion through miR-34 expression, as CCL22 also binds to CCR4 on T_REG_ cells to promote their immunosuppressive effects.^95^ Thus, targeting chemokine receptors in patients with metastatic disease might overcome immunosuppressive barriers that are established by certain immune cell populations.

### Neutralising survival factors of pro-metastatic immune cells

The colony stimulating factor (CSF) family members CSF-1, granulocyte-macrophage (GM)-CSF and granulocyte (G)-CSF are essential for the development, differentiation and survival of myeloid cells.^96^ It is perhaps then not surprising that cancer cells often directly or indirectly upregulate CSF molecules to promote pro-metastatic macrophages and neutrophils and thus to facilitate cancer progression. Consequently, targeting these molecules should reduce macrophage or neutrophil survival and negatively affect metastasis formation. For instance, early studies using *Csf1-*knockout mice, in which macrophages are severely depleted, showed that these cells are required for lung metastasis in *MMTV-PyMT* mammary tumour-bearing mice.^69^ Subsequently, antibodies and small molecules that target the CSF-1 receptor (CSF-1R) have been shown to reduce metastasis, such as in the *MMTV-HER2* mammary tumour model,^97^ and to synergise with chemotherapy.^80,98–100^ The potency of CSF-1R inhibitors has prompted many pharmaceutical companies to trial these inhibitors in cancer patients.^101^ However, anti-CSF-1R therapy has been shown to lead to increased metastasis relative to controls through an increase in the number of neutrophils mediated by a compensatory increase in serum G-CSF^102^ or a reduction in the number of NK cells as a consequence of a decrease in the myeloid-cell derived NK survival factor IL-15.^103^ These data suggest that depleting macrophages completely might not be appropriate in every scenario and that it is important to understand the nuances of macrophage biology in order to manipulate pro-metastatic polarisation states. Another point to consider when using CSF-1R inhibitors is their inability to distinguish bone marrow-derived macrophages from tissue-resident macrophages, since they have different roles in tumour development and progression and these cells may function at different stages.^104,105^ In some cases, however, such as in pancreatic cancer where bone marrow-derived macrophages play a role in antigen presentation and tissue-resident macrophages produce and remodel extracellular matrix molecules, targeting both populations might be the best approach to prevent cancer spread.^106^

G-CSF is the master regulator of granulopoiesis, and several studies have shown that inhibition of G-CSF decreases neutrophil-mediated metastasis.^75,107,108^ GM-CSF is somewhat redundant to G-CSF in neutrophil regulation; although, its expression is dominant in certain contexts.^109^ Thus, data from mouse models indicate that inhibiting G-CSF and GM-CSF in metastatic cancer patients with neutrophilia to lower neutrophil numbers may reduce secondary tumour formation and/or burden. Since chemotherapy induces neutropenia, decreased neutrophil numbers is often achieved without neutralising G-CSF or GM-CSF. Indeed, chemotherapy-induced neutropenia is associated with better outcome in patients with lung, breast, gastric and colorectal cancer,^110–113^ supporting the notion of targeting neutrophils in patients with advanced disease. However, to offset infection and neutropenia, cancer patients on chemotherapy may be given G-CSF or GM-CSF. Whether these recombinant cytokines contribute to disease progression in this context needs further investigation.

Another controversial cytokine for the potential treatment of cancer is IL-2. IL-2 is not only important for the survival and function of T_REG_ cells, which have high-affinity IL-2 receptors, but it is also vital for the activation of NK cells and effector T cells.^114^ IL-2 immunotherapy has shown limited success and severe side effects in clinical trials, which could be due to the competition of T_REG_ cells and NK cells for cytokines. However, as T_REG_ cells and NK cells express different IL-2 receptors (IL-2Rα and IL-2Rβ, respectively) efforts have been made to synthesise chimeric IL-2–IL-2Rβ or mutant IL-2 that preferentially binds to the IL-2Rβ in order to selectively activate NK cells; these agents show improved anti-tumour action, but have not been well studied in metastatic settings yet.^115,116^

### Reprogramming pro-metastatic immune cells

Because of the plasticity of myeloid cells, the metastasis-promoting phenotype of monocytes, macrophages and neutrophils can be easily influenced by tumour-derived factors. In the *MMTV-PyMT* mammary tumour model, CD4 T-cell-derived IL-4, cancer cell-derived vascular endothelial growth factor (VEGF) and endothelial cell-derived angiopoietin 2 (ANGPT2) have all been shown to modulate the phenotype of pro-metastatic macrophages to promote lung metastasis.^117–119^ In the same model, the metastasis-promoting phenotype of macrophages can be reversed epigenetically by using the class IIa histone deacetylase (HDAC) inhibitor (TMP195), which increases their phagocytic ability, reduces primary tumour burden and prevents lung metastasis.^120^ These data demonstrate that class IIa HDACs acting on macrophages may enhance the efficacy of conventional therapies in breast cancer patients. WNT signalling constitutes another pathway that drives macrophage polarisation towards a pro-metastatic phenotype. Across 16 different genetically engineered mouse models of breast cancer, WNT genes were found to be upregulated in mammary tumours driven by the loss of p53. Increased expression of WNT proteins activated macrophages to secrete IL-1β, which promoted metastasis through crosstalk with γδ T cells and neutrophils in the lung; WNT inhibition by administration of LGK974 – an inhibitor of WNT ligand secretion – re-programmed these macrophages and reduced metastasis.^77^ In some cases, however, reprogramming macrophages might not be enough to counteract metastasis, as cancer cells can harbour alternative methods to avoid destruction by macrophages. CD47, which functions as a ‘don’t eat me’ signal, can be highly expressed by cancer cells to subvert phagocytosis by signal-regulatory protein α (SIRPα)-expressing anti-tumour macrophages.^121^ CD47 is upregulated on circulating colorectal cancer cells,^122^ and its inhibition with neutralising antibodies reduces metastasis in a variety of mouse models and patient-derived xenografts.^123–128^

For many years, neutrophils were thought to be short-lived cells that were unable to respond to tumour-derived factors, but several molecules, such as G-CSF^75,107,129^ and TGF-β,^93,130,131^ have been shown to repolarise these cells towards a pro-metastatic phenotype. In p53-deficient mammary tumour models, macrophage-expressed IL-1β triggers γδ T cells to express IL-17, which induces the G-CSF-dependent expansion and polarisation of neutrophils, which, in turn, suppress the activity of cytotoxic CD8^+^ T cells.^75,77^ Consequently, inhibition of TGF-β, IL-17 and G-CSF reverses the phenotype of neutrophils and promotes the activity of cytotoxic CD8^+^ T-cells to subvert metastasis.^75,93,129,131^

Finally, as T_REG_ cells are highly abundant in tumours and suppress effector immune function, their reprogramming towards an effector phenotype could prove a fruitful strategy to increase tumour immunity and prevent metastasis. Blocking critical receptors on T_REG_ cells, such as CD25 with antibodies and genetically altering neuropilin-1 (Nrp-1), which are required to develop and maintain the stability and function of T_REG_ cells, changes these cells to pro-inflammatory cells that produce IFN-γ.^132,133^

Reprogramming any one of these immune cell populations by interfering with the cytokine cascade would, therefore, be advantageous in thwarting metastasis.

## Challenges in treating metastatic disease with immunotherapy

**Fig. 3:**
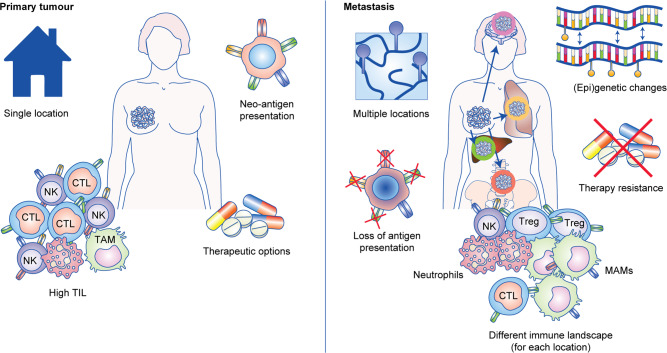
Challenges for targeting metastatic tumours. Metastatic tumours differ from primary tumours in various ways. Metastases occurring in various locations must adapt to the new tissue-specific environment (coloured circles). Metastatic tumours can acquire new (epi)genetic mutations, but antigens arising from these mutations are not always presented on the surface of cancer cells, thereby preventing T-cell recognition. The immune landscape can also be very different between primary and secondary tumours, due to varying abundance of specific immune cell populations between organs. Finally, immune responses to metastatic lesions might evolve significantly over the course of time due to acquired resistance to anti-cancer therapy (chemotherapy, radiotherapy, targeted therapy, etc) by secondary tumours.

To develop successful immunotherapies for metastatic disease, a number of considerations that might affect immunotherapy efficacy need to be taken into account. The first consideration includes the type of cancer and the location of the tumour. Although it might seem obvious, genetic mutations differ significantly between cancer types, and genetic mutations affect the immune response. Metastases can differ from their primary tumour in terms of mutational and immune profiles,^134–136^ and although there are similarities between metastases of the same organ from different cancer types, there can also be cancer-specific variations (Fig. 3). For instance, CD8^+^ T-cell infiltration is equivalent between lung metastases from colorectal cancer and renal cell carcinoma, but NK cells are more abundant and prognostic indicators in renal cell carcinoma lung metastasis.^137^ The immune contexture of primary and secondary tumours can also be analogous with regards to immunologically silent or active microenvironments as, across multiple cancer types, immune active primary tumours are more likely to generate immune active metastases.^138^ However, the immune landscape might also very well look different between primary and secondary tumours. These differences might be dependent on the organ that harbours the secondary tumour(s) or the increased mutational burden of distant metastases.^134,139–142^ Adding to this complexity, the immune landscape across metastases might not be uniform, with hot and cold tumours existing within the same patient.^134,140,143–145^

Related to this, tissue-specific immunity must also be considered, because the immune system differs between anatomical locations. Data emerging from anti-PD-1 clinical trials indicate that checkpoint inhibitors are more beneficial for patients with lung metastasis than liver metastasis, for example.^146–149^ In melanoma patients, liver metastases have a lower density of CD8^+^ T cells at the tumour margin when compared with metastases in other organs,^149^ which could explain the reduced response to PD-1 inhibition at this site. Likewise, in patients with metastatic prostate cancer, anti-CTLA-4 induces anti-tumour T_H_1 cell-type CD4^+^ T-cell responses in primary tumours, but this same response is absent in bone metastatic lesions. Instead, the bone marrow tumour microenvironment, which is rich in TGF-β, converts CD4^+^ T cells into T_H_17 cells to blunt anti-CTLA-4 immunotherapy at this site.^150^ Combining TGF-β inhibitors with anti-CTLA-4 to generate T_H_1 CD4^+^ T cell and CD8^+^ T-cell responses can reverse these effects in metastatic lesions. Brain and bone metastasis represent some of the most challenging tumours to treat, largely due to aberrant vascularisation at these sites or their immune-specialised status. Bone is a particularly immune-privileged site in order to protect and preserve the hematopoietic stem cell compartment. In breast cancer bone metastasis, the cycle of bone degradation and tumour growth has been shown to be a critical event in permitting the outgrowth of metastatic cells from dormancy.^151^ Therefore, combining immunotherapies with osteoclast inhibitors may be key in the success of treating bone metastasis. In the case of brain metastasis, the success of T cell and NK cell-based immunotherapies is heavily dependent on antibodies or small molecules being able to penetrate the blood brain barrier or reactivate T cells in highly immunosuppressive cervical lymph nodes.^152^ Recently, it has emerged that anti-PD-1 and anti-CTLA-4 therapy in tandem significantly increased intracranial anti-tumour activity in patients with metastatic melanoma, offering promise for the use of immune checkpoints in treating brain metastasis.^153^ However, modulation of T-cell trafficking molecules and/or dendritic cell assistance may be critical to overcome immunosuppression of T cells and NK cells in brain and bone metastasis.

Finally, the success of T-cell- and NK cell-based immunotherapy is dependent on the ability of these immune cells to recognise cancer cells. For example, HLA loss of heterozygosity (LOH) precludes the presentation of (neo)antigens by cancer cells, and HLA LOH can be more common in metastatic tumours than in primary tumours.^134,154,155^ Dormant cancer cells—non-proliferating malignant cells hiding in distant organs—are another challenge in immune-cell recognition,^156^ as MHC-I expression might be downregulated on these cells.^157^ In addition to the ability of T cells to recognise cancer cells, the other components of the cancer immunity cycle—including antigen release, antigen presentation, T-cell priming, trafficking, tumour infiltration and killing—must be intact in patients for favourable outcomes of T-cell-based therapies.^158^ If any of these components are missing or become inactive as a result of cancer evolution, T-cell-based immunotherapies fail or become inadequate at controlling metastatic lesions. Moreover, acquired resistance to T-cell- and NK cell-based immunotherapy may arise after initially providing a strong anti-tumour response. Some of the mechanisms of acquired resistance have been identified, including HLA loss as stated above,^134,154,155^ epigenetic dysfunction of T-cells,^159^ emergence of additional immunosuppressive pathways,^160^ or resistance to IFN-γ via new cancer-specific mutations.^161,162^ Thus, inherent and acquired resistance to T-cell- and NK cell-based immunotherapy is a major challenge.

To overcome these challenges, a deeper understanding of the immune landscape, genetic mutations, components of the cancer immunity cycle and tissue-specific immunity is needed to facilitate personalised approaches to immunotherapy in metastatic disease. What is clear from experimental models and on-going clinical trials is that the immune response to metastatic lesions can change dramatically over time, and it is not linear. Therefore, future treatment modalities will need to anticipate how pro-tumour and anti-tumour immune cells react to first line immunotherapies and mitigate roadblocks with additional immunomodulatory drugs.

## Conclusions

As outlined in this article, a great many new immunotherapeutic targets are on the horizon for metastatic disease. Possibly, however, the biggest improvement for patients will come from the use of combination therapies that both boost anti-tumour immunity and attenuate immunosuppression. Biomarkers arising from the study of anti-PD-1/CTLA-4 non-responding patients or from those patients who acquire resistance might also identify suitable immunotherapy targets to re-engage anti-tumour immune activity when anti-PD-1/CTLA-4 approaches become inert. Mechanisms to induce tertiary lymphoid structures and antigen-presenting B cells—two anti-tumour features not well explored in metastasis—could support effector T-cell responses and complement immunotherapies in metastatic disease, as seen in patients with melanoma or sarcoma.^163–165^

To optimally exploit these immunotherapies, it will be important to increase our understanding of the context in which they are most efficacious. These efforts will require more knowledge regarding the interplay between specific molecules and specific cell types in the metastatic setting. Choosing the right model is paramount to address this knowledge gap. Injectable cell lines, such as the B16 melanoma cells, have been instrumental in immunotherapy discovery, but they fail to represent the full metastatic cascade. The immunotherapy field will need to adopt or create models that recapitulate the evolution of the immune response that occurs when tumours are allowed to progress from early stage to late stage metastatic disease. These models might help to uncover immunotherapy targets that specifically rewire the pre-metastatic niche, prevent cancer cell seeding or eliminate established tumours at distant sites. Combining metastasis models with humanised mice might also be useful to enhance personalised immunotherapies. It is hoped that the development of these tools will generate new insights into immunotherapeutic intervention for metastatic disease.

## Data Availability

Not applicable.
